# Characterization of NAC transcription factor NtNAC028 as a regulator of leaf senescence and stress responses

**DOI:** 10.3389/fpls.2022.941026

**Published:** 2022-08-15

**Authors:** Lichao Wen, Tao Liu, Zhichao Deng, Zenglin Zhang, Qi Wang, Weifeng Wang, Wei Li, Yongfeng Guo

**Affiliations:** Tobacco Research Institute, Chinese Academy of Agricultural Sciences, Qingdao, Shandong, China

**Keywords:** tobacco, *NtNAC028*, abiotic stress, leaf senescence, ROS, *Arabidopsis thaliana*

## Abstract

NAC proteins constitute one of the largest transcription factor families and are involved in regulation of plant development and stress responses. Our previous transcriptome analyses of tobacco revealed a significant increase in the expression of *NtNAC028* during leaf yellowing. In this study, we found that *NtNAC028* was rapidly upregulated in response to high salinity, dehydration, and abscisic acid (ABA) stresses, suggesting a vital role of this gene in abiotic stress response. *NtNAC028* loss-of-function tobacco plants generated *via* CRISPR-Cas9 showed delayed leaf senescence and increased tolerance to drought and salt stresses. Meanwhile *NtNAC028* overexpression led to precocious leaf senescence and hypersensitivity to abiotic stresses in *Arabidopsis*, indicating that *NtNAC028* functions as a positive regulator of natural leaf senescence and a negative regulator of stress tolerance. Furthermore, *NtNAC028*-overexpressing *Arabidopsis* plants showed lower antioxidant enzyme activities, higher reactive oxygen species (ROS), and H_2_O_2_ accumulation under high salinity, resulted in more severe oxidative damage after salt stress treatments. On the other hand, *NtNAC028* mutation in tobacco resulted in upregulated expression of ROS-scavenging and abiotic stress-related genes, higher antioxidant enzyme activities, and enhanced tolerance against abiotic stresses, suggesting that *NtNAC028* might act as a vital regulator for plant stress response likely by mediating ROS scavenging ability. Collectively, our results indicated that the *NtNAC028* plays a key regulatory role in leaf senescence and response to multiple abiotic stresses.

## Introduction

Plants are sessile organisms that must withstand and cope with environmental pressures, including biotic and abiotic stresses. Adverse changes in environmental conditions usually cause loss of crop yield, even the death of plant tissues (Liliane and Charles, 2020). Abiotic stresses, including drought and salinity, pose threats to food security worldwide by causing significant loss of crop production (Moghaddam et al., 2021). These stresses often lead to excessive accumulation of reactive oxygen species (ROS), which disrupts cellular redox homeostasis and causes oxidative damage of plant cells (Suzuki et al., 2012). In order to survive adverse environments, plants have developed a series of biochemical and physiological strategies during evolution (Haak et al., 2017). Gene regulation at the transcriptional level is the most critical and effective way for plants’ adaptation to stresses and DNA-binding transcription factors (TFs) play a vital role in this process (Lata et al., 2011; Le Hénanff et al., 2013). To date, extensive studies have revealed a wide range of TFs including MYB, bZIP, WRKY, and NAC (NAM, ATAF1/2, and CUC2) that are involved in regulation of abiotic stress response (Guo and Gan, 2012; Pascual et al., 2018; Zhang et al., 2018).

NAC (NAM, ATAF1/2, and CUC2) proteins are one of the largest families of plant-specific TFs, most of which harbor a highly conserved DNA-binding domain at the N-terminal region and a variable transcriptional regulation C-terminal domain (Diao et al., 2020). NAC TFs have been implicated in multiple aspects of plant development. For example, CUP-SHAPED COTYLEDON1 (CUC1) and NAC WITH TRANSMEMBRANE MOTIF1 (NTM1) are involved in shoot and lateral root development. NAC SECONDARY WALL THICKENING PROMOTING FACTOR1 (NST1) and VASCULAR-RELATED NAC DOMAIN6 (VND6) are master regulators of secondary cell wall formation, ORESARA9 (ORE9) and NAC-LIKE, ACTIVATED BY AP3/PI (AtNAP) play important role in regulating leaf senescence (Takada et al., 2001; Guo and Gan, 2006; Kim et al., 2006; Zhong et al., 2010; Breeze et al., 2011; Diao et al., 2020; Liu et al., 2022). Besides, NAC proteins have also been reported to be implicated in different abiotic stress response (Nakashima et al., 2012). A number of NAC proteins involved in regulating stress response have been characterized and manipulated to develop crop varieties with enhanced stress tolerance. In *Arabidopsis*, expression of *ANAC019*, *ANAC072/RD26*, *ANAC053/NTL4*, or *ANAC055* could improve salt and drought tolerance of transgenic plants (Tran et al., 2004; Lee et al., 2014). *anac096* mutant is drought stress sensitive, indicating that ANAC096 functions as a positive regulator in dehydration stress response (Xu et al., 2013). Besides, ANAC016 could also positively regulate drought stress tolerance through suppressing *AREB1* expression (Sakuraba et al., 2015). In contrast, *ORE1* knockout mutants are more tolerant to salt stress, while overexpression lines show an opposite phenotype, suggesting that ORE1/ANAC092 functions as a negative regulator in salt stress response (Balazadeh et al., 2010). In rice, overexpression of *OsNAC5*, *OsNAC6*/*SNAC2*, *OsNAC45*, *OsNAC52*, or *OsNAC066* significantly enhanced the tolerance to drought and salt stresses in transgenic rice (Nakashima et al., 2007; Sperotto et al., 2009; Gao et al., 2010; Yuan et al., 2019; Zhang et al., 2020). For wheat, *TaNAC2*, *TaNAC67*, and *TaNAC29* have been reported to participate in abiotic stress responses. Overexpression of these genes in Arabidopsis and wheat improved plants’ tolerance to low temperature, high salinity, and drought by activating stress-responsive genes (Mao et al., 2012, 2014, 2016; Huang et al., 2015). Similarly, overexpression of several maize NACs, including *ZmSNAC1*, *ZmNAC33*, *ZmNAC49*, and *ZmNAC55*, could confer tolerance to drought stress in transgenic plants (Lu et al., 2012; Mao et al., 2016; Liu et al., 2019; Xiang et al., 2021). Interestingly, many stress-related genes might also be involved in plant senescence process. For instance, OsNAC2, known to negatively regulate many abiotic stresses tolerances, can also promote rice leaf senescence *via* ABA biosynthesis (Shen et al., 2017). TaNAC29 has been reported to play crucial roles in senescence and abiotic stress response (Huang et al., 2015; Xu et al., 2015). It has been hypothesized that some of these genes integrate the different signaling pathways and play important roles in the crosstalk between stress responses and age-dependent senescence (He et al., 2005; Guo and Gan, 2006).

Compared with *Arabidopsis thaliana*, *Oryza sativa*, and other plant species, few studies have been conducted on the functional characteristics of tobacco *NAC* genes. *NtNAC-R1* has been reported to be involved in nicotine synthesis and lateral root development (Fu et al., 2013). Besides, overexpression of *NtNAC2* or *NtNAC053* could improve drought and/or salt stress tolerance in tobacco plants (Xu et al., 2018; Li et al., 2022). Our previous study has shown that overexpression of *NtNAC080* promotes leaf senescence in *Arabidopsis*, suggesting that it acts as a positive regulator of natural leaf senescence in tobacco (Li et al., 2018). A recent study has indicated that NaNAC29 may participate in the defense responses to *Alternaria alternata* and promote leaf senescence in *Nicotiana attenuata* (Ma et al., 2020). To our knowledge, no further studies have been conducted on stress-related *NAC* genes in tobacco.

We previously identified a NAC TF, NtNAC028 (accession number XM_016636746), as a senescence upregulated gene. In this work, we found that *NtNAC028* was also dramatically induced by high salinity, dehydration, and ABA treatments, suggesting that this gene might have multiple functions in different biological processes. Then we performed a detailed functional characterization of *NtNAC028* in abiotic stress responses using transgenic tobacco and *Arabidopsis*. Phenotypic analysis showed that NtNAC028 played a negative role in response to drought and salt stress. Further study revealed that the enhanced tolerance of *ntnac028* tobacco mutant obtained *via* CRISPR-Cas9 strategy was potentially achieved by activation of antioxidant enzymes in ROS scavenging and upregulation of stress-responsive genes. In addition to being involved in abiotic stress responses, NtNAC028 could also positively regulate leaf senescence in tobacco. Our findings demonstrated the role of NtNAC028 in leaf senescence, drought and salt stress responses and revealed the likely molecular mechanisms underlying abiotic stress response in tobacco.

## Materials and methods

### Plant growth conditions and stress treatments

Common tobacco (*Nicotiana tabacum* L. Cv. K326) seeds were sterilized by 70% ethanol for 1 min and 15% w/v H_2_O_2_ for 12 min, then rinsed in sterile water 4–5 times. The seeds were germinated and the plants were grown on Murashige and Skoog (MS) medium in a plant growth chamber (Conviron, Canada) at 25°C under long-day conditions (16 h light/8 dark). Then 4-week-old seedlings were removed from MS plates and treated in MS solution in 250 ml beakers with or without 200 mM NaCl, 150 μM abscisic acid (ABA), 150 μM salicylic acid (SA), or 150 μM jasmonic acid (JA) for 0–36 h. For drought treatments, 4-week-old seedlings were removed from MS medium, placed on Whatman No.5 filter paper, and dehydrated at room temperature for 0, 1, 3, 6, and 12 h. Whole plants were harvested, immediately frozen in liquid nitrogen, and stored at-80°C before further use. Three biological replicates were applied for each treatment.

*Arabidopsis* (Col-0) plants were grown on soil or on plates in a growth chamber at 22°C with hours a 16-h-light/8-h-dark cycle. For growth on plates, seeds were surface sterilized with 70% ethanol for 5 min, washed 4–5 times with sterile water, and then stored in the dark at 4°C for 3 days before the plates were transferred to growth chamber.

### RNA isolation and quantitative real-time PCR analysis

Total RNA was extracted using the Ultrapure RNA kit (CW biotech, Beijing, China), and first-strand cDNAs were synthesized using the HiScript III All-in-one RT SuperMix (Vazyme, NanJing, China). Then the cDNA samples were used for qRT-PCR analysis, which was performed on a Light Cycler96 real-time PCR system (Roche, Germany). *NtActin* and *AtActin* were used as internal reference genes in tobacco and *Arabidopsis*, respectively. Three technical replicates were used for all reactions and three biological replicates were performed for each experiment. The relative expression values were calculated using the 2^-△△Ct^ method (Rao et al., 2013). The relevant primers are listed in Supplementary Table 1.

### Generation of transgenic *Arabidopsis* plants

The full-length *NtNAC028* cDNA was amplified by PCR using primes (*NtNAC028*-35 s-F: 5′-CACTGT TGATACATATGGTTGGGAAAAATTGCTCCGAG-3′; *NtNAC028*-35 s-R: 5′-TGTTGATTCAGAATTCTCACTGGA ACTGAAAGGCTGGAT-3′) and cloned into linearized *pRI101-AN* vector (digested with *NdeI* and *EcoRI*) using the Infusion kit (Clontech, Bejing, China). The recombinant vector was transferred into *Agrobacterium tumefaciens* GV3101 and then transformed into *Arabidopsis thaliana* (Col-0) *via* the floral dip method (Clough and Bent, 1998). The transgenic plants were selected on 1/2 MS medium supplemented with 50 mg/ml kanamycin and confirmed by qRT-PCR. Homozygous T_3_ lines were used for senescence and stress response assays.

### Generation of *NtNAC028* knockout tobacco *via* CRISPR-Cas9 genome editing

For CRISPR-Cas9 vector construction, an online software^1^ was used to design the guide RNA (gRNA) targeting the *NtNAC028* coding region. The forward and reverse primers were annealed to form DNA double-strands and inserted into the *pORE-Cas9* vector (Gao et al., 2015). The recombinant vector was introduced into *Agrobacterium tumefaciens* LBA4404 and transformed into common tobacco (K326) by *Agrobacterium*-mediated transformation (Horsch et al., 1989). The transgenic plants were screened by kanamycin (50 mg/ml) resistance and used for extraction of genomic DNA. For each transgenic plant, the region of the gRNA targeting site was amplified by PCR using primers (*NtNAC028*-TS-F: 5′-ATGGTTGGGAAAAATTGCT-3′; *NtNAC028*-TS-R: 5′-GATTGCTTTATCTGTGCCTGT-3′) and cloned into the *pEASY-blunt* Zero vector (Transgene, China). DNAs from single colonies were used for sequencing to detect the mutation type. T_1_ plants, derived from self-pollinated T_0_ mutant lines, were sequenced again to confirm mutations. The homozygous mutants were further analyzed.

### Measurements of chlorophyll content and Fv/Fm

The extraction and quantification of chlorophyll were performed as previously described (He and Gan, 2002). Briefly, 200 mg of fresh leaves was extracted with 5 ml of anhydrous ethanol in dark until the chlorophyll was completely dissolved. The supernatant was measured at 665 and 649 nm for absorbance. For determination of Fv/Fm, after 30 min of dark treatment, the leaves of individual plant were determined using a IMAGING-PAM M-series Chlorophyll Fluorescence System (LI-6400-40 LCF, Walz, Effeltrich, Germany). Chlorophyll contents and Fv/Fm were calculated and mean values from three biological replicates were obtained.

### Dark/NaCl-induced senescence

Leaf disks from 60-day-old plants of the *ntnac028* mutant and WT were placed onto moistened filter paper inside Petri dishes, and kept in dark for 7 days. Three independent biological replicates of this experiment were carried out. For NaCl-induced senescence assay, leaf disks from 35-day-old plants were placed in dishes with 0 or 200 mM NaCl, and kept in light for 7 days. Similarly, the leaves of 4-week-old plants from *NtNAC028*-OE *Arabidopsis* lines and WT (Col-0) plants were placed in dishes with 0 or 200 mM NaCl, and kept in light for 3 days. Three independent biological replicates of this experiment were carried out.

### Drought and salt stress tolerance assays in planta

For the drought tolerance assays, WT (Col-0) and *NtNAC028*-OE *Arabidopsis* lines were soil-grown for 4 weeks under well-watered conditions, then stressed by withholding water for 8 days. After that, the plants were re-watered and survival rate of each group of plants was calculated after 3 days. Similarly, 5-week-old plants of the *ntnac028* mutant and WT (K326) were subject to dehydration by halting water for 12 days, and then re-watered for 2 days. After that, the survival rates of tobacco seedlings were calculated. The water loss rate was measured by calculating the weight change of detached leaves of different genotypes after dehydration for 2 h at room temperature.

For salt stress treatment, the surface-sterilized tobacco seeds grown on MS medium were placed vertically in a growth chamber for 2 weeks and transferred to MS medium supplemented with 0, 50, 100, 150, or 200 mM NaCl, respectively. The plants were then grown for 2 weeks before measuring the length of primary roots. Similarly, 4-day-old seedlings of different *Arabidopsis* lines were transferred into 1/2 MS medium supplemented with 100 or 150 mM NaCl, respectively. After 1 week, the length of the primary roots was measured.

For germination assay, surface-sterilized seeds of *NtNAC028*-OE and WT were sown on 1/2 MS medium supplemented with different concentrations of NaCl (0, 100, and 150 mM) or ABA (0.5 μM), and kept at 4°C for 3 days in the dark before being transferred to a growth chamber under normal growth conditions. After 1 week, the germination rates of each group were calculated based on the presence of open cotyledons. All the experiments were performed with three independent biological replicates.

### Measurements of MDA and antioxidant enzyme activities

The content of malondialdehyde (MDA) was determined as previously described (Draper et al., 1993). POD, SOD, and CAT enzymatic activities were measured using the detection kits provided by Beijing Solarbio Science & Technology Co., Ltd.

### Determination of H_2_O_2_ accumulation

For NBT staining, the salt-treated leaves from *NtNAC028*-OE lines and WT were soaked in NBT staining buffer (10 mM potassium phosphate buffer, pH 7.2) for 12 h. After that, leaf chlorophyll was removed by a mixed solution (glycerol/acetic acid/ethanol, 1:1:3) and then stored in a solution consisting of ethanol/glycerol (4:1). The hydrogen peroxide content was determined using a hydrogen peroxide assay kit (Solarbio, China) according to the manufacturer’s instruction.

## Results

### *NtNAC028* expression is upregulated by multiple stresses

**Figure 1:**
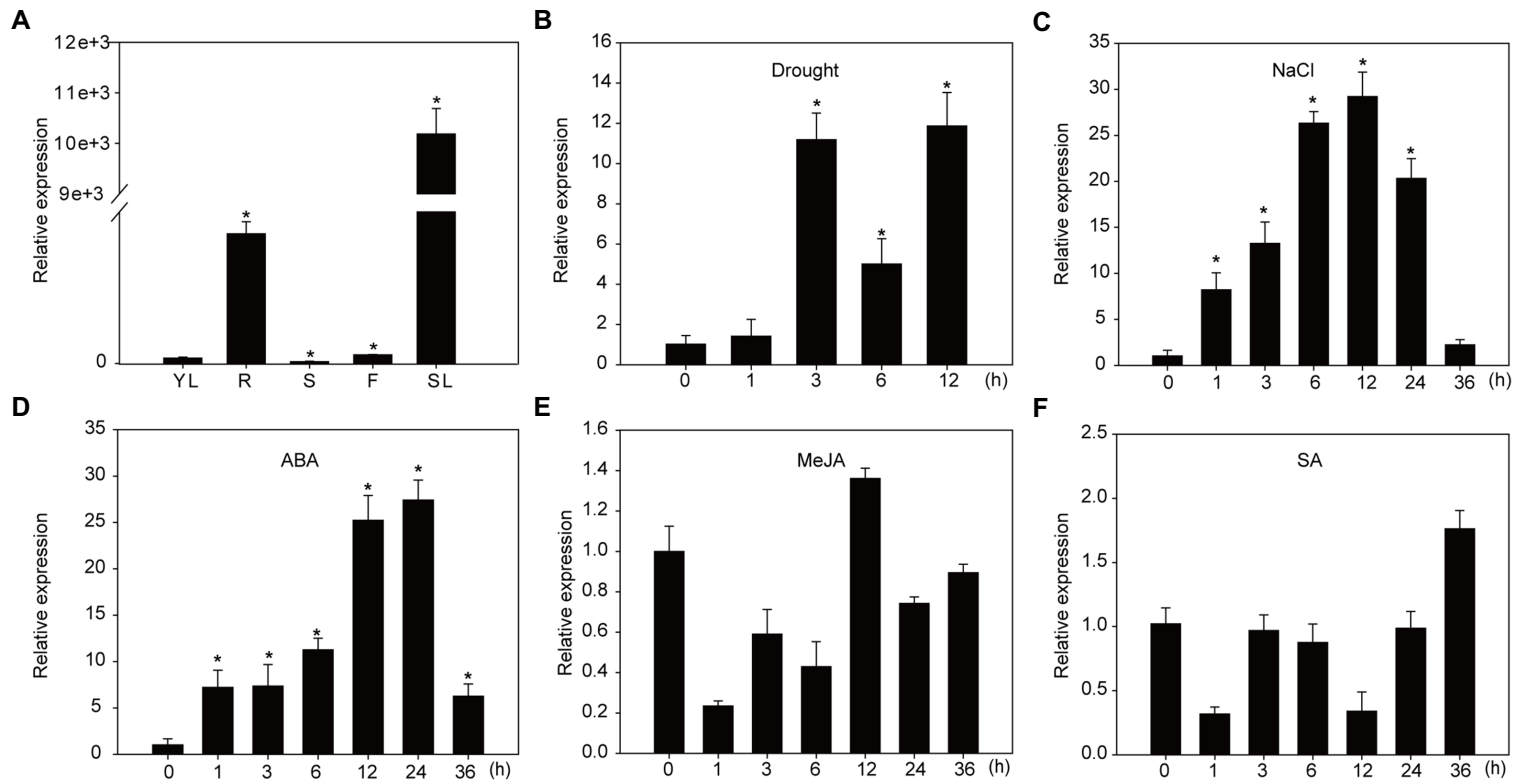
Expression profiling of *NtNAC028*. **(A)** The expression pattern of *NtNAC028* in various organs of tobacco plants (K326). YL, young leaf; R, root; S, stem; F, flower; SL, senescence leaf. **(B,C)** *NtNAC028* expression in response to different stress conditions. **(D–F)** The expression pattern of *NtNAC028* under exogenous phytohormone treatments. ABA, abscisic acid; JA, jasmonic acid; and SA, salicylic acid. The bars are SD of three biological replicates. Asterisks indicate significant difference by Student’s *t*-test (^*^p < 0.05).

Our previous work showed that the expression of *NtNAC028* increased significantly during tobacco leaf senescence, especially at the early stage of senescence (Li et al., 2018). In this study, qRT-PCR was performed to measure the expression of *NtNAC028* in tobacco. Among the tested tissues, the transcript level of *NtNAC028* was the highest in the senescent leaves and very low in young leaves and stems (Figure 1A). In addition, *NtNAC028* showed high expression in roots, suggesting that *NtNAC028* might have multiple biological functions. To understand whether *NtNAC028* is also involved in abiotic stress response, we examined the expression patterns of *NtNAC028* under salt and drought stress treatments. As shown in Figures 1B,C, the transcript level of *NtNAC028* was induced significantly during the whole course (1–24 h) of these treatments. Meanwhile, the expression patterns of *NtNAC028* after exogenous plant hormone treatment were analyzed. The expression of *NtNAC028* increased continuously and rapidly after ABA treatments. However, no significant increase in *NtNAC028* expression was observed after treatments with SA or JA (Figures 1D–F).

### *NtNAC028* positively regulates leaf senescence

**Figure 2:**
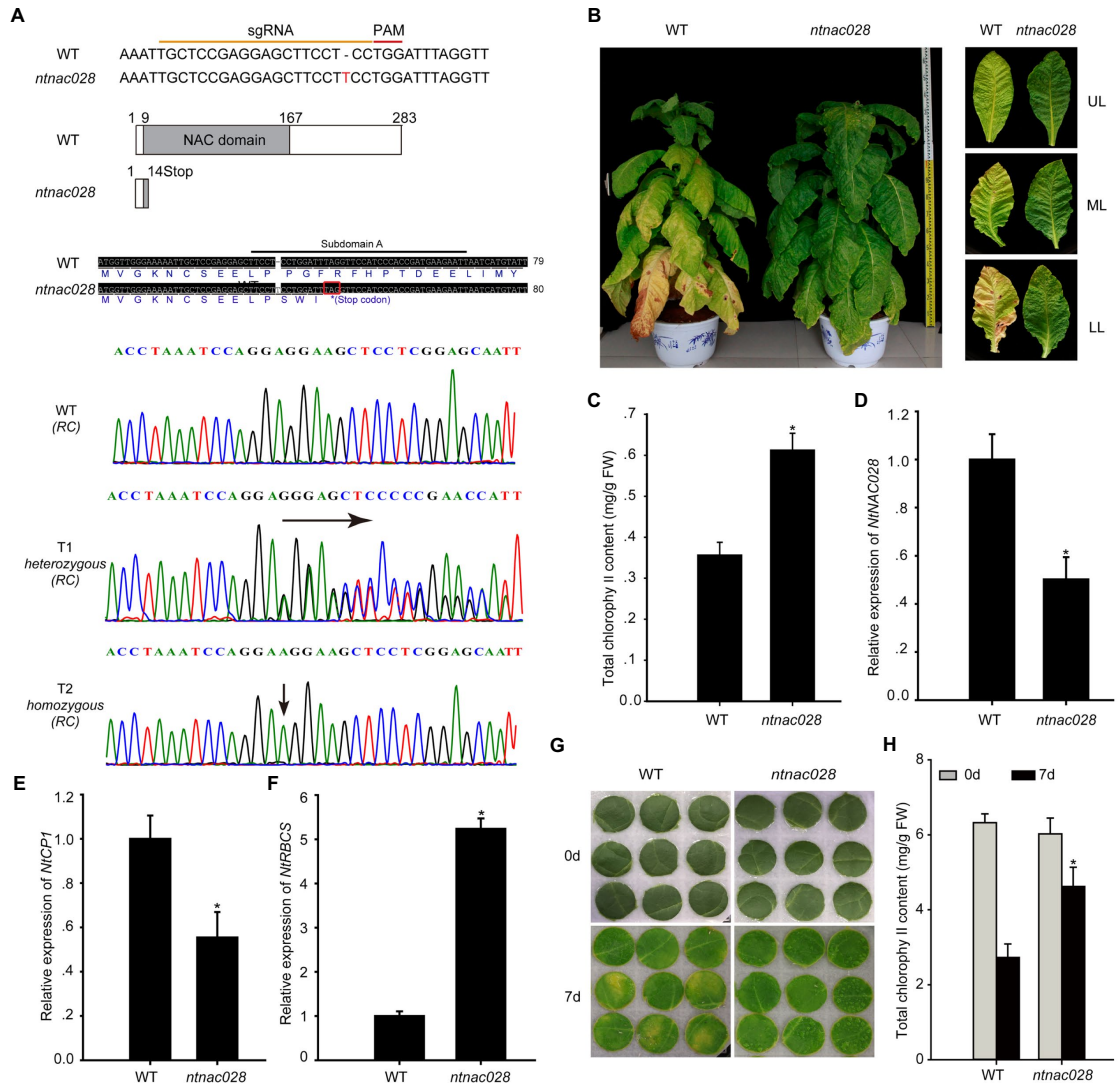
*NtNAC028* positively regulates leaf senescence. **(A)** Construction of the *NtNAC028* CRISPR-Cas9 vector. Sanger sequencing results showed an insert of 1 bp in the genomic DNA of *ntnac028*, compared to the WT. Red line represents the PAM sequence. **(B)** Leaf senescence phenotypes of *ntnac028*. **(C)** Chlorophyll content of middle leaves in age-matched wild-type (K326) and *ntnac028* plants. UL (upper leaf), ML (middle leaf), and LL (low leaf). **(D–F)** Expression of *NtNAC028, NtCP1, and NtRBCS* in middle leaves of WT and *ntnac028*. **(G)** Phenotypes of detached leaves under dark conditions. **(H)** Chlorophyll content of detached leaves in WT and *ntnac028* under darkness for 7 days. The bars are SD of three biological replicates. Asterisks indicate statistically significant difference from WT (^*^p < 0.05).

To understand the role of *NtNAC028* in regulating tobacco leaf senescence, we generated a *NtNAC028* loss-of-function line using the CRISPR-Cas9 system. A gRNA targeting the NAC domain of *NtNAC028* was designed and used for *Agrobacterium*-mediated transformation of tobacco plants. T_0_ transgenic plants were obtained after kanamycin selection and sequenced to identify mutations. Then homozygous mutant plants were obtained from T_1_ generation for further phenotypic analysis (Figure 2A). Here, the *ntnac028* mutant had a 1 bp insertion at the 3′ end of the gRNA sequence which introduced a stop codon after position P14 and led to a truncation of the DNA-binding NAC domain and loss of the transcriptional regulation domain at the C-terminus (Figure 2A). Compared with WT (K326), the *ntnac028* mutant plants showed a delayed leaf senescence phenotype. The chlorophyll content of middle leaves from the *ntnac028* mutant were higher than that in WT (Figures 2B,C). qRT-PCR showed that the transcript level of *NtNAC028* in the mutant was significantly lower than WT (Figure 2D), possibly due to decreased stability of the truncated coding region. Furthermore, the transcript levels of senescence-related genes were determined by qRT-PCR. Compared to WT, the expression level of *CYSTEINE PROTEINASE 1* (*NtCP1*, *SENESCENCE-ASSOCIATED GENE 12/SAG12* homolog in tobacco) was lower and the *RIBULOSE BISPHOSPHATE CARBOXYLASE SMALL CHAIN* (*NtRBCS*) was higher in the *ntnac028* mutant (Figures 2E,F). Likewise, we investigated the phenotype of the *ntnac028* mutant leaves under dark treatments (Figure 2G). Detached leaves of WT and *ntnac028* plants were covered with aluminum foil for 7 days. Leaves of the *ntnac028* mutant showed a delayed senescence phenotype compared with those from WT. Consistent with this finding, the leaves of *ntnac028* mutant had higher chlorophyll content compared with WT (Figure 2H). These data suggested that *NtNAC028* could act as a positive regulator in tobacco leaf senescence.

To further evaluate the role of *NtNAC028* in regulating leaf senescence, we generated transgenic *Arabidopsis* plants overexpressing *NtNAC028*. 45 days after sowing, the overexpression transgenic plants, with expression of *NtNAC028* confirmed by qRT-PCR, showed precocious leaf senescence (Supplementary Figures S1A,B). The chlorophyll contents and maximum quantum efficiency of PSII (Fv/Fm) of *NtNAC028*-OE plants (the fifth leaves) were lower than that of WT (Supplementary Figures S1C,D). Moreover, the premature senescence phenotype was also supported by the higher expression of *AtSAG12* (a senescence marker gene) in the fifth leaves of the two *NtNAC028* OE lines (Supplementary Figure S1E). These results also indicated that *NtNAC028* could positively regulate leaf senescence.

### CRISPR/Cas9-mediated knockout of *NtNAC028* enhances abiotic stress tolerance in tobacco

**Figure 3:**
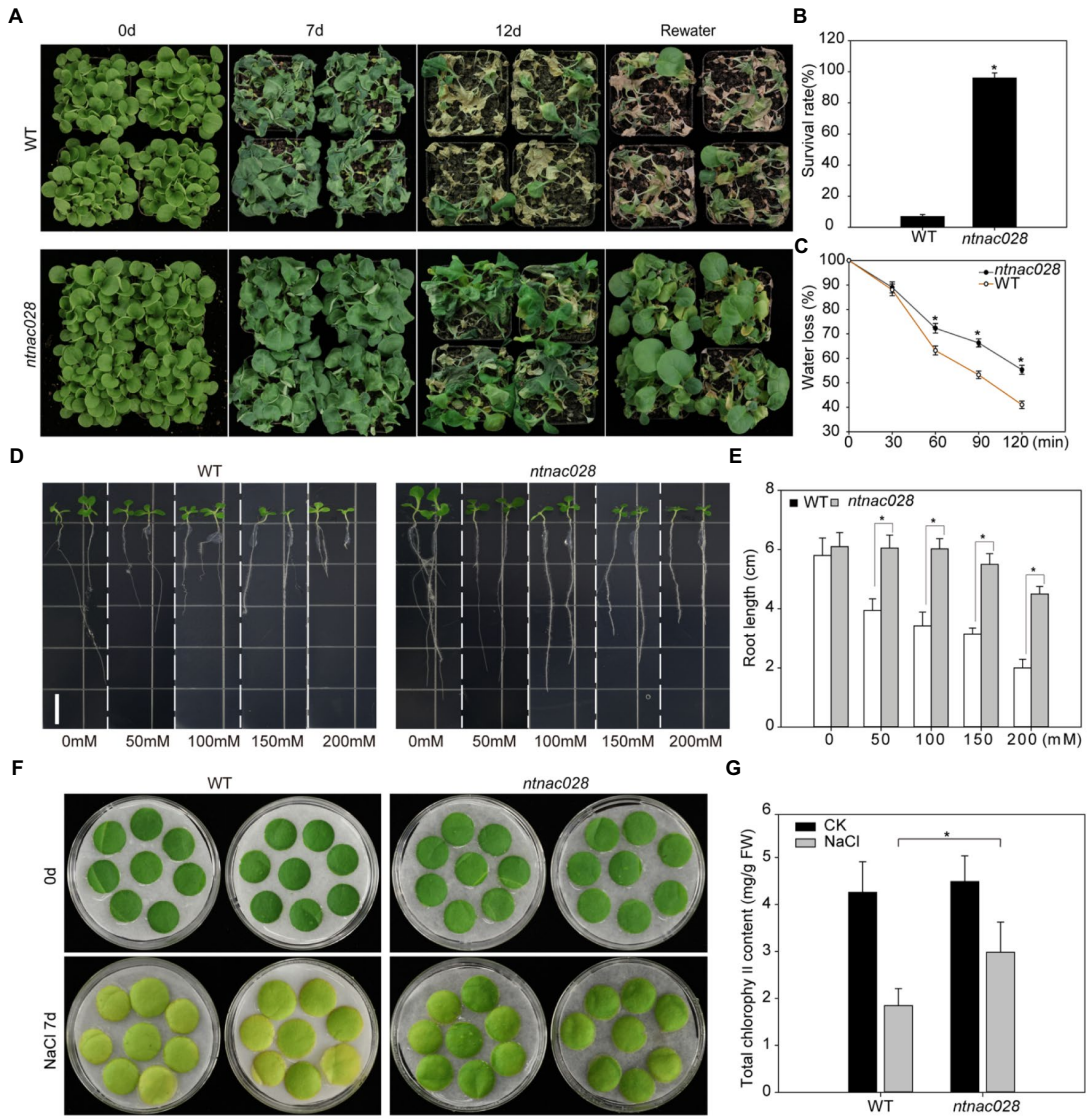
CRISPR/Cas9-mediated knockout of *NtNAC028* enhances abiotic stress tolerance in tobacco. **(A)** Phenotypes of tobacco plants treated with drought stress for 12 days and recovered with water for 2 days. **(B)** Phenotypes of *ntnac028* and wild-type tobacco under drought stress. **(C)** Water loss rates of detached leaves from WT and *ntnac028*. Water loss was determined as the percentage of initial fresh weight. Values are means from 10 plants for each of three independent experiments. **(D,E)** Root length of WT and *ntnac028* under control and NaCl treatments. Seedlings were vertically cultivated on MS plates containing 0, 50, 100, 150, or 200 mM NaCl. Images were taken after 5 days. Bars = 1 cm. Data were calculated using 20 seedlings for each genotype. The bars are SDs of three biological replicates. Asterisks indicate statistically significant difference from WT (*p < 0.05). **(F,G)** The phenotypes and chlorophyll content of detached leaves from WT and *ntnac028* under NaCl treatment for 7 days.

In order to examine the biological role of NtNAC028 in response to drought stress, 5-week-old *ntnac028* and WT tobacco plants were subject to drought stress treatments. As shown in Figure 3A, the *ntnac028* plants displayed higher drought tolerance. Around 7 days after water withdraw, when most leaves of the WT plants were wilted, only a small portion of the *ntnac028* leaves started wilting. Most of the WT plants became desiccated 12 days after drought treatment and died after re-watering, with a survival rate of 6.9%. The *ntnac028* plants on the other hand, behaved much better at the end of the treatment with a survival rate of 96% after re-watering (Figures 3A,B). Detached leaves of the *ntnac028* mutant and WT (K326) were subject to dehydration stress for 2 h and water loss rate was calculated at each time point. During the first 30 min of dehydration stress, there was no significant difference in relative water content between *ntnac028* and WT plants. However, the relative water content of *ntnac028* plants was significantly higher than that of WT after 1 h of dehydration stress. After 2 h dehydration stress, the water content of WT was less than 42%, while it was still more than 55% in the *ntnac028* mutant (Figure 3C). This result was consistent with the enhanced drought tolerance in the *ntnac028* mutant. Besides, we also analyzed the root length of *ntnac028* and WT seedlings grown under treatments with different concentrations of NaCl. There was no significant difference in root length between *ntnac028* plants and WT under normal conditions. However, in the presence of various concentrations of NaCl, the *ntnac028* plants were more tolerant than WT. The root length of the *ntnac028* plants was dramatically longer than that of WT (Figures 3D,E). For the germination assay, there was no significant difference between *ntnac028* and WT plants in the absence of NaCl. However, the germination rates of *ntnac028* seeds treated with 50 or 100 mM NaCl were significantly higher than that of WT plants, indicating that the seeds of *ntnac028* mutant were insensitive to NaCl during the germination stage (Supplementary Figures S2A,B). Furthermore, detached leaves from 5-week-old plants of *ntnac028* and WT were treated with 200 mM NaCl for 7 days. The leaves of WT senesced early with lower levels of chlorophyll content than *ntnac028* leaves under NaCl stress conditions (Figures 3F,G). Taken together, these results indicated that the knockout of *NtNAC028* led to elevated abiotic stress tolerance in tobacco.

### Overexpression of *NtNAC028* confers hypersensitivity to abiotic stress in transgenic *Arabidopsis*

**Figure 4:**
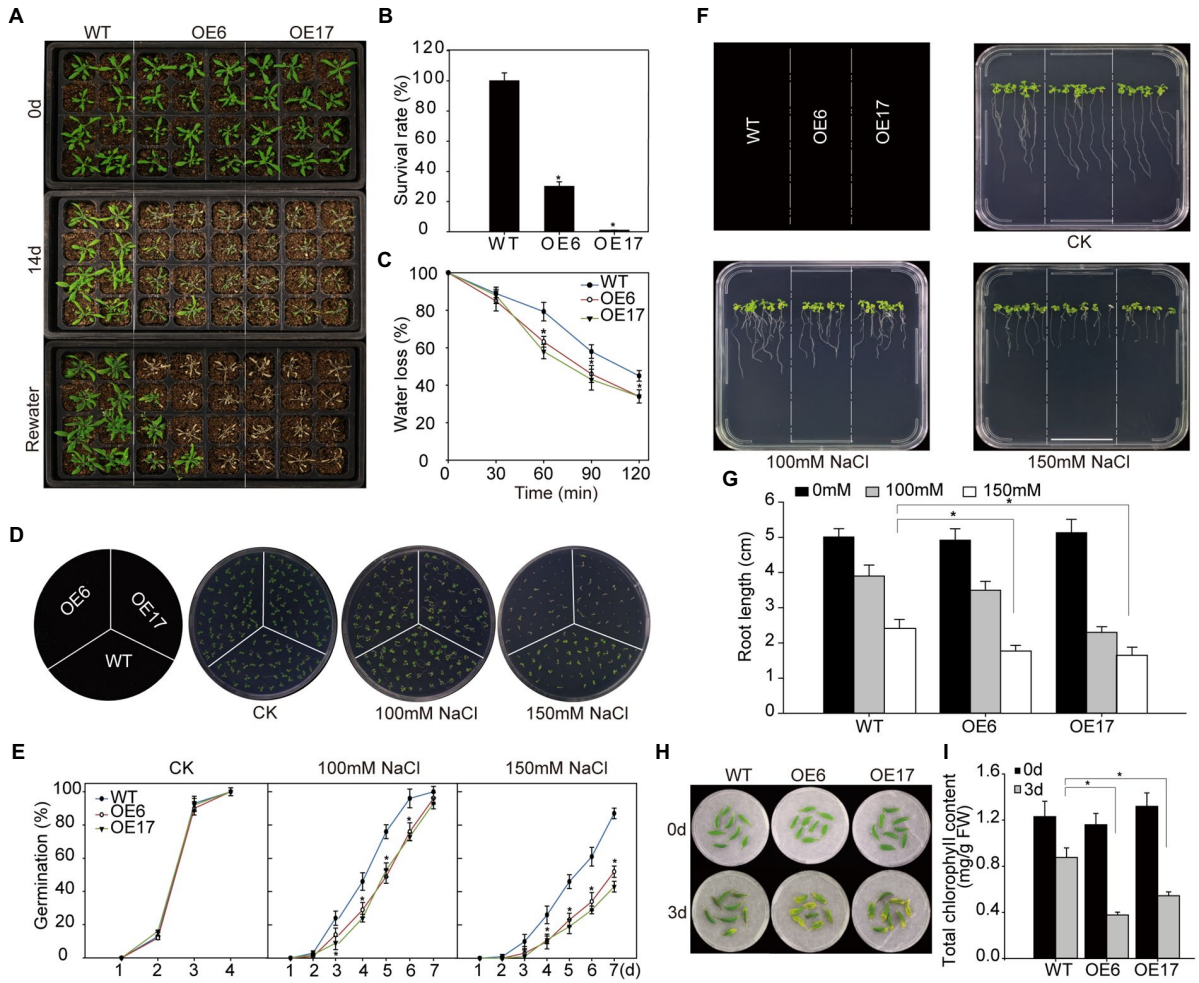
Overexpression of *NtNAC028* confers hypersensitivity to abiotic stress in transgenic *Arabidopsis*. **(A)** Phenotypes of *NtNAC028* overexpression lines (OE6 and OE17) and WT under drought stress treatments. Four-week-old *NtNAC028* overexpression lines and WT were withheld from watering for 14 days, followed by re-watering and recovery for 3 days. **(B)** Survival rates of *NtNAC028* overexpression lines and WT after drought stress. **(C)** Water loss rates of detached leaves from *NtNAC028* overexpression lines and WT. **(D,E)** Germination of WT and *NtNAC028* overexpression lines with different concentrations of NaCl as indicated. Representative images were taken after 7 days. **(F,G)** Root length of *NtNAC028* overexpression lines and WT seedlings under NaCl treatments. Seedlings were vertically grown for 7 days on 1/2 MS plates containing 0, 100, or 150 mM NaCl. **(H,I)** The phenotypes and chlorophyll content of detached leaves from WT and NtNAC028 overexpression lines under NaCl treatments for 3 days. The bars are standard deviations (SD) of three biological replicates. Asterisks indicate statistically significant difference from WT (^*^p < 0.05).

To evaluate the effect of *NtNAC028* overexpression on drought stress tolerance, soil-grown *Arabidopsis* plants were subject to drought stress by withholding water. After the dehydration stress for 8 days, *NtNAC028*-OE6 and *NtNAC028*-OE17 plants exhibited severe leaf wilting phenotypes due to water deficiency, while no evident wilting symptom was observed for WT plants (Figure 4A). After re-watering for 3 days, less than 35% of the overexpression plants survived; whereas, the survival rates of WT plants were close to 100% (Figure 4B). Moreover, *NtNAC028*-OE lines lost more water than the WT plants at all time points in the water loss assay of detached leaves (Figure 4C). These results indicated that overexpression of *NtNAC028* reduced drought tolerance in *Arabidopsis*.

Meanwhile, *NtNAC028*-OE plants were examined for tolerance to salt stress. As shown in Figures 4D,E, no obvious difference in germination rate was observed without NaCl treatment. However, germination rates of *NtNAC028*-OE lines were significantly lower than that of WT in the presence of 100 or 150 mM NaCl. This result indicated that NaCl stress could severely inhibited seed germination of *NtNAC028* overexpression lines compared to WT. Meanwhile, the germination assay was carried out to assess the ABA sensitivity of *NtNAC028*-OE lines. Interestingly, the *NtNAC028*-OE lines had significantly lower germination rates than WT under ABA treatments (Supplementary Figures S3A,B), suggesting that *NtNAC028*-OE seeds were more sensitive to ABA during germination. Besides, the sensitivity to salt in *NtNAC028* overexpression lines was further assayed at the post-germination stage. Similarly, no significant difference in root length between *NtNAC028*-OE lines and WT was observed under the control conditions. In the presence of 100 or 150 mM NaCl, the roots of the *NtNAC028*-OE lines were significantly shorter than that of WT plants (Figures 4F,G). Furthermore, detached leaves from 4-week-old plants of *NtNAC028*-OE and WT were treated with 200 mM NaCl for 3 days. The leaves of *NtNAC028*-OE senesced early with lower levels of chlorophyll content than WT leaves under NaCl stress conditions (Figures 4H,I). Overall, these results suggested that overexpression of *NtNAC028* reduced salt tolerance in *Arabidopsis*.

### *NtNAC028* overexpression increases ROS accumulation and regulates expression of stress-responsive genes under abiotic stress treatments

**Figure 5:**
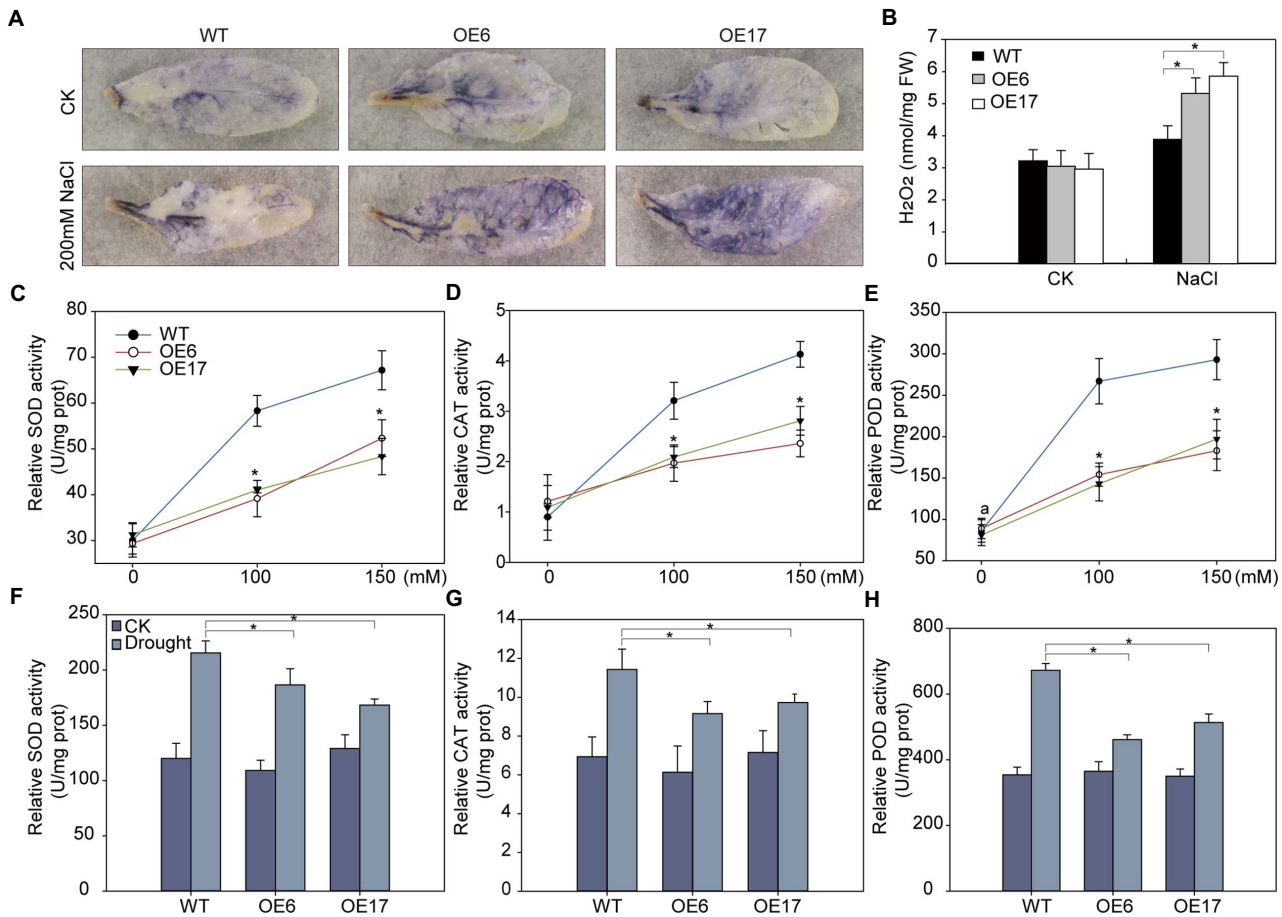
*NtNAC028* promotes reactive oxygen species (ROS) accumulation under NaCl treatments. **(A)** NBT staining. The middle leaves of 4-week-old *NtNAC028* overexpression lines and WT with or without NaCl treatments were used for NBT staining. **(B)** Measurement of accumulation of H_2_O_2_ in detached leaves of plants with different genotypes. **(C–H)** POD, SOD, and CAT activities of *NtNAC028* overexpression lines and WT after salt and drought stress treatments. The bars are standard deviations (SD) of three biological replicates. Asterisks indicate statistically significant difference from WT (^*^p < 0.05).

Drought or salt stress often leads to oxidative stress which involves excessive ROS generation in plant cells (Choudhury et al., 2017). To explore whether the reduced tolerance to drought and salt in *NtNAC028*-OE lines is associated with the alteration of ROS levels, we used NBT staining to visualize the levels of superoxide radical (O^.2−^) in leaves. Without NaCl treatment, the color of NBT staining in leaves showed no significant difference between *NtNAC028*-OE and WT plants. When stressed by 200 mM NaCl, the *NtNAC028*-OE lines accumulated significantly more O^.2−^, as densely stained with dark blue color in the leaves of *NtNAC028*-OE lines compared to WT (Figure 5A). Besides, we measured the endogenous H_2_O_2_ levels of plants with different genotypes. In the presence of NaCl stress, *NtNAC028*-OE plants accumulated significantly more H_2_O_2_ than that of WT (Figure 5B). These results indicated that *NtNAC028* overexpression could enhance ROS production in transgenic *Arabidopsis* under salt stress treatments.

Furtherly, we analyzed the activities of SOD, POD, and CAT, which are main enzymes responsible for scavenging ROS. No obvious difference was observed in the ROS scavenging activities under non-stress conditions. After salt and drought treatments, SOD, POD, and CAT activities of *NtNAC028*-OE lines and WT were all significantly increased. However, the increase in enzymatic activities of the *NtNAC028*-OE lines was significantly less than that of WT (Figures 5C–H). Furthermore, the expression levels of ROS-scavenging-related genes (SOD, POD, and CAT) in *NtNAC028*-OE lines were also lower than that of WT plants after salt and drought treatments (Supplementary Figures S4A–F). The expression patterns of stress-responsive genes including *AtRD29A, AtDREB1B, AtRD26, and AtNCED3-2* were analyzed in *NtNAC028*-OE lines and WT plants before and after drought/salt treatments (Supplementary Figures S5A–H). Under no-stressed conditions, no significant difference in the transcript levels of these stress-related genes was observed between *NtNAC028*-OE and WT plants. However, the expression levels of these gene were significantly lower in *NtNAC028*-OE lines than in WT under drought or salt treatments. These results suggested that *NtNAC028*-OE could reduce the expression levels of ROS scavenging-related and stress-responsive genes upon abiotic stresses.

### *NtNAC028* mutation enhances the antioxidant activity of tobacco under abiotic stresses

**Figure 6:**
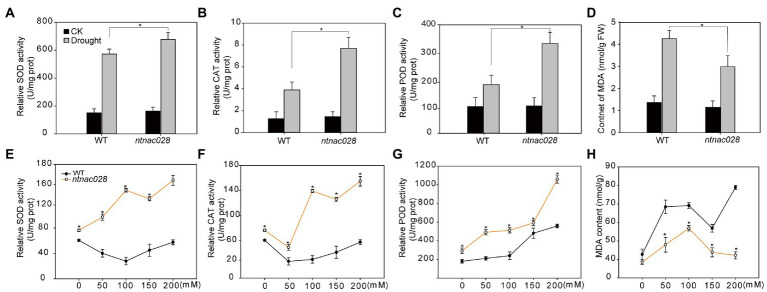
*NtNAC028* mutation enhances the antioxidant activity of tobacco under abiotic stresses. **(A–C)** POD, SOD, and CAT activities of WT and *ntnac028* plants with or without drought stress treatments. **(E–G)** POD, SOD, and CAT activities of WT and *ntnac028* plants under various concentrations of NaCl. **(D,H)** MDA contents of WT and *ntnac028* plants under drought and salt treatments. The bars are standard deviations (SD) of three biological replicates. Asterisks indicate statistically significant difference from WT (*p < 0.05).

To ascertain whether the enhanced stress tolerance of *ntnac028* plants was associated with the alteration of ROS scavenging capability, we examined the activities of SOD, POD, and CAT of *ntnac028* and WT plants under normal or stressed conditions. No significant difference was observed in activities of the antioxidant enzymes under non-stress conditions. However, the activities of SOD, POD, and CAT were more dramatically increased in *ntnac028* plants than that in WT after drought or salt stress treatments (Figures 6A–C,E). Consistently, MDA content of *ntnac028* plants was significantly lower than that in WT following drought/salt stress treatments (Figures 6D,H). These results indicated that NtNAC028 mutation could enhance stress tolerance possibly through enhancing the activities of antioxidant enzymes.

### NtNAC028 regulates the expression of scavenging-related and stress-responsive genes in tobacco under abiotic stresses

**Figure 7:**
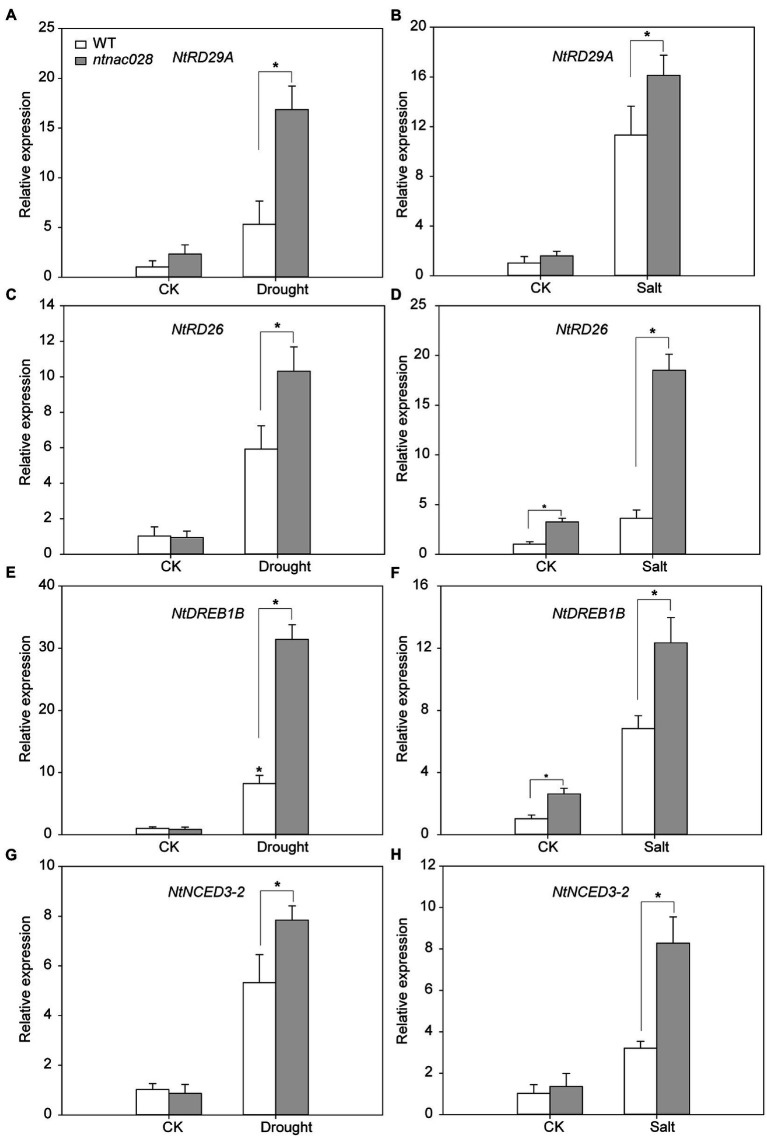
Expression patterns of stress-responsive genes in WT and *ntnac028* plants under salt and drought treatments. Relative transcript levels of four stress-responsive genes (RD29A, RD26, DREB1B, and NCED3-2) in WT and *ntnac028* plants before and after drought **(A,C,E,G)** and salt **(B,D,F,H)** treatments. The bars are standard deviations (SD) of three biological replicates. Asterisks indicate statistically significant difference from WT (*p < 0.05).

To understand the mechanism of NtNAC028’s regulation of abiotic stress response, the expression patterns of stress-responsive genes, including *NtRD29A*, *NtDREB1B*, *NtRD26*, and *NtNCED3-2* were analyzed in *ntnac028* and WT plants before and after drought/salt treatments (Figures 7A–H). Under no-stressed conditions, no significant difference in the transcript levels of these stress-related genes was observed between *ntnac028* and WT plants. However, the expression levels of these genes were significantly higher in *ntnac028* than in WT plants under drought or salt treatments. Furthermore, salt and drought treatments strongly induced the expression of ROS-scavenging-related genes (SOD, POD, and CAT) in *ntnac028* compared with WT (Supplementary Figures S6A–F). These results suggested that *NtNAC028* knockout could enhance the expression levels of ROS scavenging-related and stress-responsive genes upon abiotic stresses.

## Discussion

The NAC superfamily is one of the largest transcription factor families in plants, which plays important regulatory roles in plant development and stress response (Forlani et al., 2021). Previously, we identified a total of 154 NAC genes (*NtNACs*) in tobacco and demonstrated that NtNAC080 was a positive regulator of leaf senescence (Li et al., 2018). However, the functions of most of the remaining NtNACs remain unclear. In this study, we carried out a detailed functional characterization of NtNAC028, which belongs to the same phylogenetic subgroup as NtNAC080. The results demonstrated that NtNAC028 could also positively regulate leaf senescence, sharing a redundant role with NtNAC080. In addition, the *NtNAC028* transcript was found to be upregulated rapidly and significantly under drought, salinity, and exogenous ABA treatments, suggesting it may be involved in abiotic stress response. Previous studies have revealed that some members of the NAC TFs are involved in senescence and also play vital roles in stress response (Guo and Gan, 2012; Zhang and Gan, 2012). For instance, overexpression of *TaNAC29* from wheat can enhance salt and drought tolerance in transgenic *Arabidopsis*, which exhibits a delayed senescence phenotype (Huang et al., 2015). It was also showed that AtNAP negatively regulates tolerance to osmotic stress, while positively regulates leaf and fruit senescence (Guo and Gan, 2006; Xiaohong Kousup Sup, 2012). Interestingly, OsNAP, a rice homologue of AtNAP, functions as a positive regulator of leaf senescence and abiotic stress responses (high salinity and drought; Liang et al., 2014). In addition to having the similar function as positive regulators of leaf senescence, NAP homologues in different plant species seem to have more diversified functions in stress responses. In this study, several lines of evidence indicated that *NtNAC028* plays an important role in response to drought and high salinity. As expected, the promoter sequence of *NtNAC028* (about 3 kb upstream of the transcription start site) contains multiple cis-related elements of plant stress response, including seven ABA-responsive elements (ABREs) and three stress-related elements (TC-rich repeats: defense and stress responsiveness; LTR: low-temperature responsiveness; Supplementary Table 2). These results indicated that *NtNAC028* might have broad functions in abiotic response.

NAC TFs are known to be involved in environmental stresses, which in turn improve the ability of plants in tolerating these stresses (Nakashima et al., 2012). Previous studies have showed that abiotic stresses could induce premature leaf senescence, which accelerates dry matter accumulation in grains and plays a positive role in plant environmental adaptability (Sade et al., 2018). While the mutants with delayed leaf senescence often showed higher tolerance to stress treatments (Kang et al., 2019; Guo et al., 2021). Here, *ntnac028* tobacco mutant generated by CRISPR/Cas9 showed a lower rate of water loss and enhanced salt and drought stress tolerance, while *NtNAC028*-OE *Arabidopsis* lines were more sensitive to these stress than WT. In addition, seed germination rates of *NtNAC028*-OE lines were much lower than that of WT under NaCl treatments. These results suggested that NtNAC028 might negatively regulate salt and drought stress responses. It is known that the responsiveness of plants to abiotic stresses was mediated by ABA-dependent and ABA-independent pathways. In the ntnac028 mutant, the ABA-dependent pathway genes *NtRD26, NtRD29A, and NtNCED3-2*, as well as the ABA-independent gene *NtDREB1B*, were expressed at a higher level under stress conditions. It has been reported that these genes are involved in the mediation of oxidative or osmotic damages induced by stresses (Wei et al., 2016; Huang et al., 2018; Jiang et al., 2019; Orbović et al., 2021). For example, RD26 acts as a transcriptional activator of ABA-inducible gene expression in plants under various abiotic stress conditions (Fujita et al., 2004). DREB1B is a member of the AP2/ERF family, which can activate a subset of downstream stress-responsive genes and plays an essential role in drought stress response (Wei et al., 2016). Thus, increased expression of these genes could help *ntnac028* mutant plants in tolerating abiotic stresses. As being reported in our previous study, NtNAC028 is a homolog of AtNAP (Li et al., 2018). AtNAP has been documented to negatively regulate salt stress tolerance *via* repression of ABA-dependent stress-responsive genes (Seok et al., 2017), suggesting that NtNAC028 might perform similar regulatory function in abiotic stress response in tobacco.

It is well known that abiotic stresses could cause excessive accumulation of reactive oxygen species (ROS) in plants, which could lead to damage of cellular biomolecules, including proteins, lipids, and DNA (Parida and Das, 2005). In addition, the senescence process can also induce ROS generation, and the increase in ROS levels triggers and accelerates senescence (Maggiorani et al., 2017). Therefore, ROS may serve as a key signal in both senescence and stress responses. Besides, antioxidant enzymes, such as POD, SOD, and CAT are the enzymes responsible for scavenging ROS in plants (Gill and Tuteja, 2010; Miller et al., 2010). The genes encoding antioxidant enzymes are often upregulated to protect tissues under stresses (Jin et al., 2017). In the present study, *NtNAC028*-OE lines accumulated significantly higher levels of H_2_O_2_ than WT plants under salt stress conditions. The activities of POD, CAT, and SOD in *NtNAC028*-OE lines were also significantly lower than those of WT (Figure 5), indicating that the higher accumulation of ROS in *NtNAC028*-OE plants under stress conditions was due to their inability to remove ROS in time. In contrast, the transcript levels and activities of POD, SOD, and CAT in the *ntnac028* mutant were significantly higher than that in WT (K326; Figures 6, 7). Consistently, MDA content of *ntnac028* mutant plants was significantly lower than that of WT under stress conditions, indicating that the *ntnac028* mutant plants produced less ROS. Thus, it is possible that a more efficient ROS scavenging system in the *ntnac028* mutant is important for its abiotic stress tolerance.

## Conclusion

In summary, we functionally characterized a tobacco NAC gene, *NtNAC028*, which was strongly induced by senescence and various abiotic stresses. *NtNAC028* mutation induced by CRISPR/Cas9 led to delayed senescence and enhanced tolerance to drought and salinity in tobacco, while *NtNAC028* overexpression transgenic *Arabidopsis* exhibited early senescence and hypersensitivity to high salinity and drought, indicating that NtNAC028 culd function as a negative regulator of leaf senescence and abiotic stress response in plants. Furthermore, our results revealed that the enhanced stress tolerance of the *ntnac028* mutant might be due to enhanced scavenging capacity of ROS and increased expression of stress-responsive genes.

## Data availability statement

The original contributions presented in the study are included in the article/Supplementary material, further inquiries can be directed to the corresponding authors.

## Author contributions

YG and WL conceived the research. WL and LW generated the transgenic materials and performed all experiments, and drafted the manuscript. TL, ZD, and QW assisted in data collection and analysis. ZZ and WW designed the Crispr-Cas9 experiment and generated the constructs. All authors contributed to the article and approved the submitted version.

## Funding

This work was financially supported by National Natural Science Foundation of China (No. 31970204), the Agricultural Science and Technology Innovation Program (ASTIP-TRIC02)and Funds for Special Projects of the Central Government in Guidance of Local Science and Technology Development (21-1-1-1-zyyd-nsh).

## Conflict of interest

The authors declare that the research was conducted in the absence of any commercial or financial relationships that could be construed as a potential conflict of interest.

## Publisher’s note

All claims expressed in this article are solely those of the authors and do not necessarily represent those of their affiliated organizations, or those of the publisher, the editors and the reviewers. Any product that may be evaluated in this article, or claim that may be made by its manufacturer, is not guaranteed or endorsed by the publisher.

## Footnotes

^1^ http://www.multicrispr.net/index.html

## Supplementary material

Supplementary Figure S1 Overexpression of NtNAC028 in *Arabidopsis* plants causes precocious senescence.

Supplementary Figure S2 Seed germination under salt treatments.

Supplementary Figure S3 Seed germination under ABA treatments.

Supplementary Figure S4 Expression patterns of reactive oxidation species scavenging-related genes in WT and NtNAC028-OE plants in response to salt and drought treatments.

Supplementary Figure S5 Expression patterns of stress responsive genes in WT and NtNAC028-OE plants under salt and drought treatments.

Supplementary Figure S6 Expression patterns of ROS scavenging-related genes in WT and ntnac028 plants in response to salt and drought treatments.
