# Supplementary figures and images for: Characterization of NAC transcription factor NtNAC028 as a regulator of leaf senescence and stress responses
Source: Front Plant Sci. 2022 Aug 15;13:941026. doi: 10.3389/fpls.2022.941026 (PMC9421438; doi:10.3389/fpls.2022.941026)

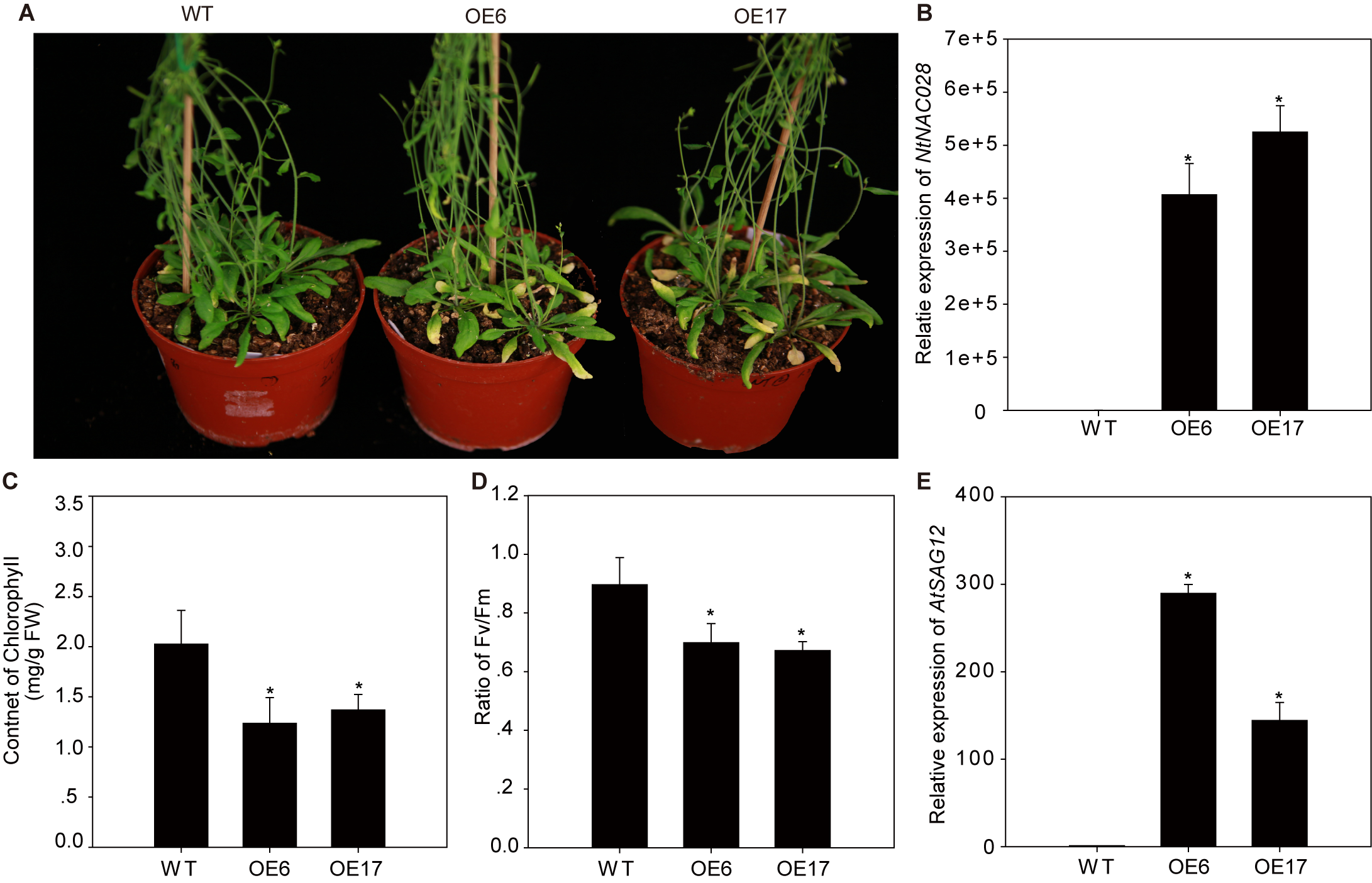

Supplement: Supplementary Figure S1 — Overexpression of NtNAC028 in Arabidopsis plants causes precocious senescence. [file Image_1.TIF]

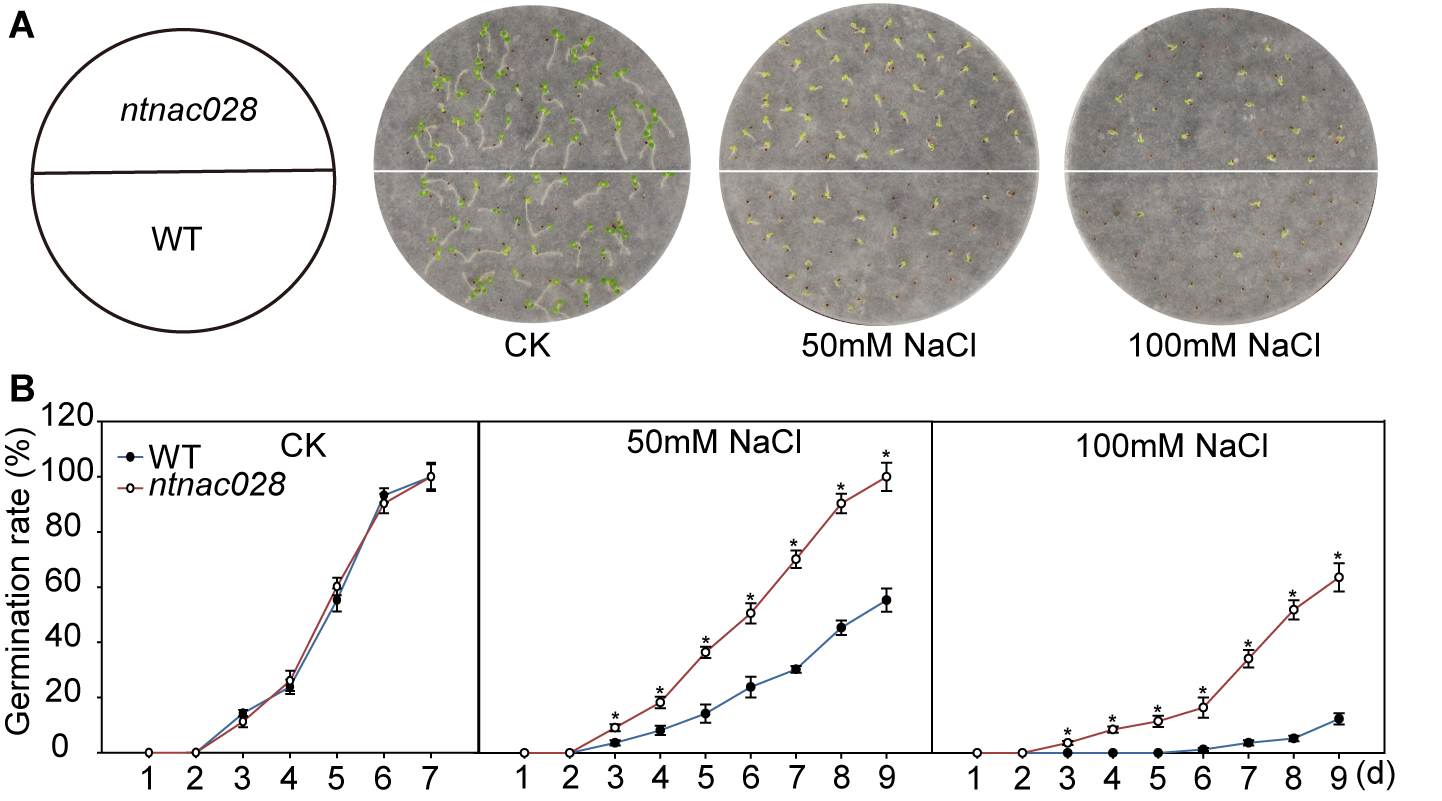

Supplement: Supplementary Figure S2 — Seed germination under salt treatments. [file Image_2.TIF]

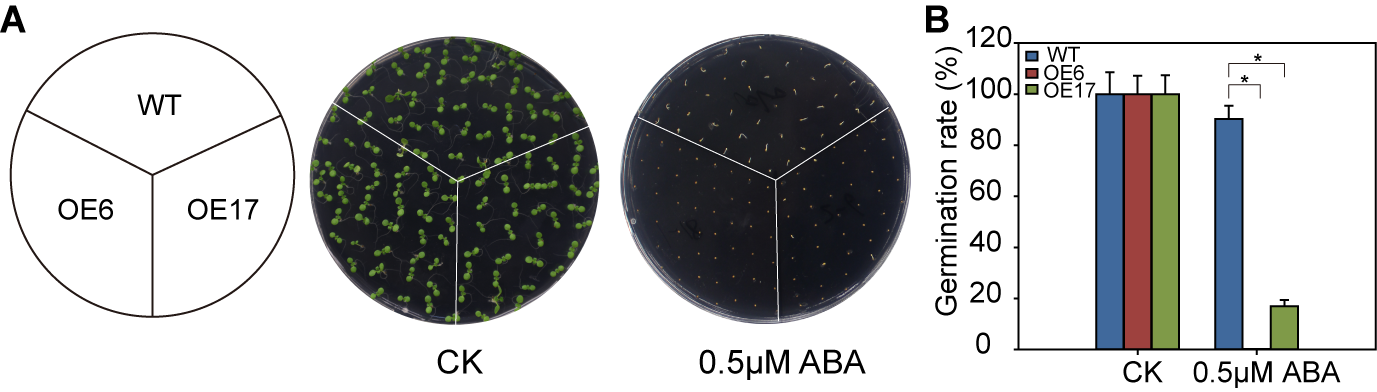

Supplement: Supplementary Figure S3 — Seed germination under ABA treatments. [file Image_3.TIF]

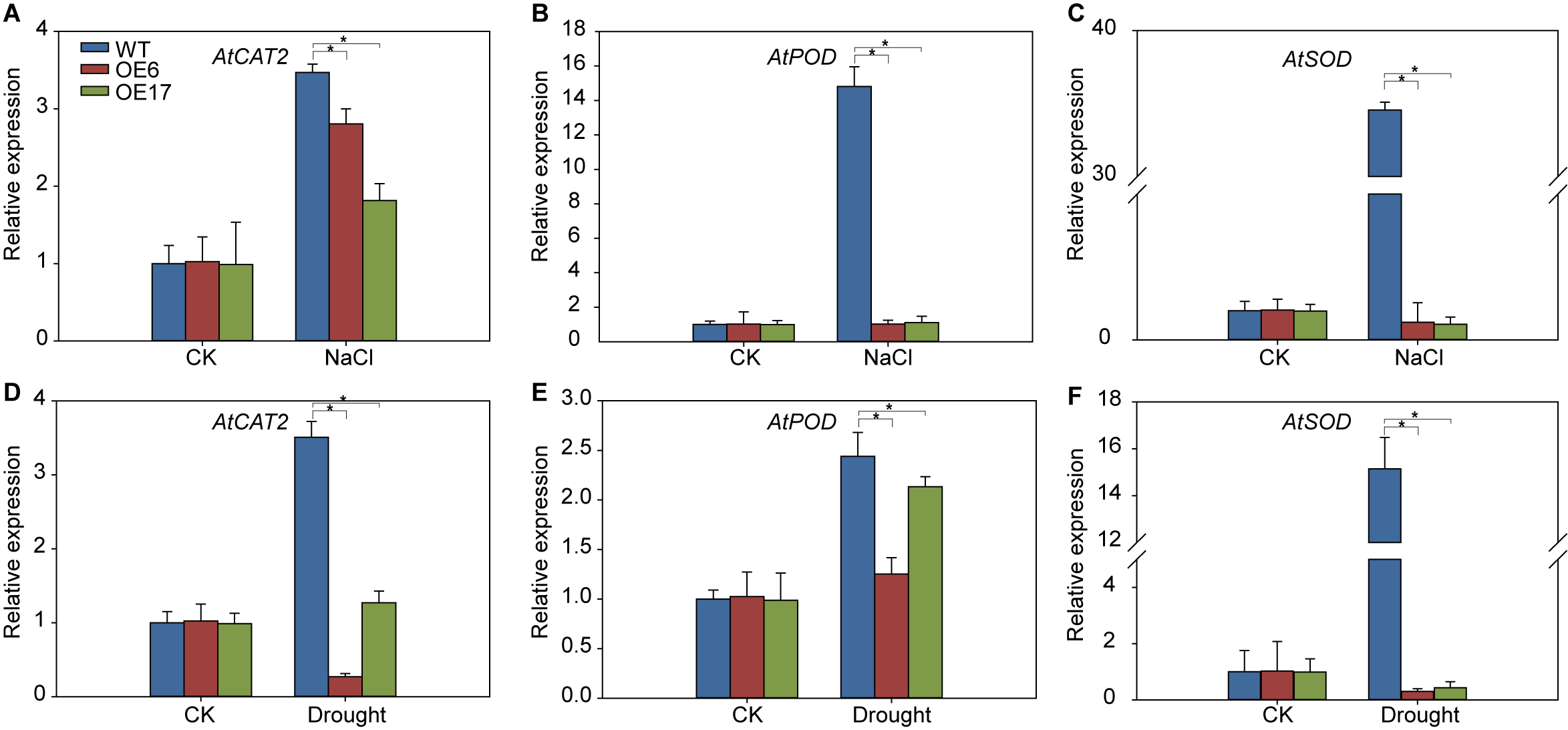

Supplement: Supplementary Figure S4 — Expression patterns of reactive oxidation species scavenging-related genes in WT and NtNAC028-OE plants in response to salt and drought treatments. [file Image_4.TIF]

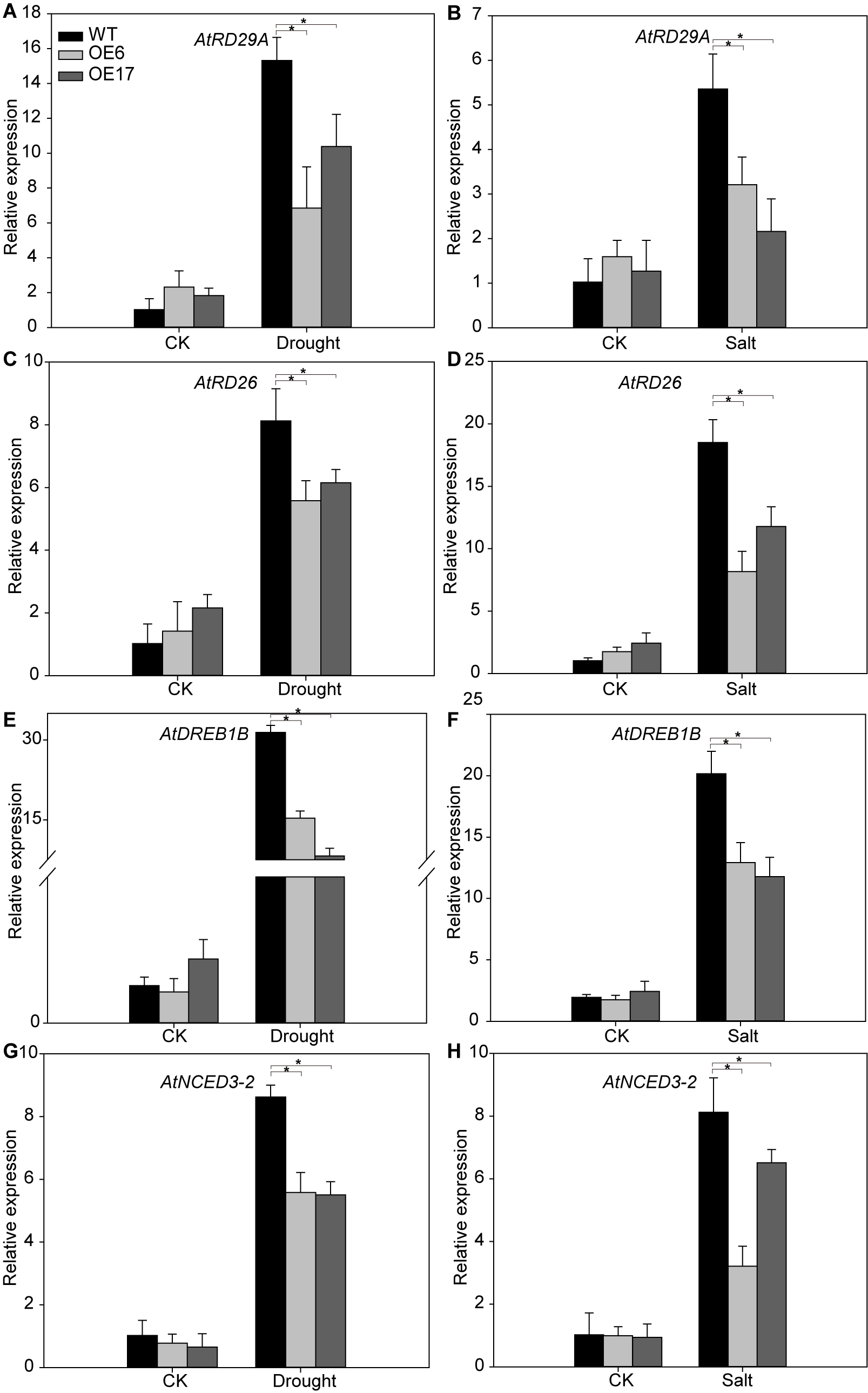

Supplement: Supplementary Figure S5 — Expression patterns of stress responsive genes in WT and NtNAC028-OE plants under salt and drought treatments. [file Image_5.TIF]

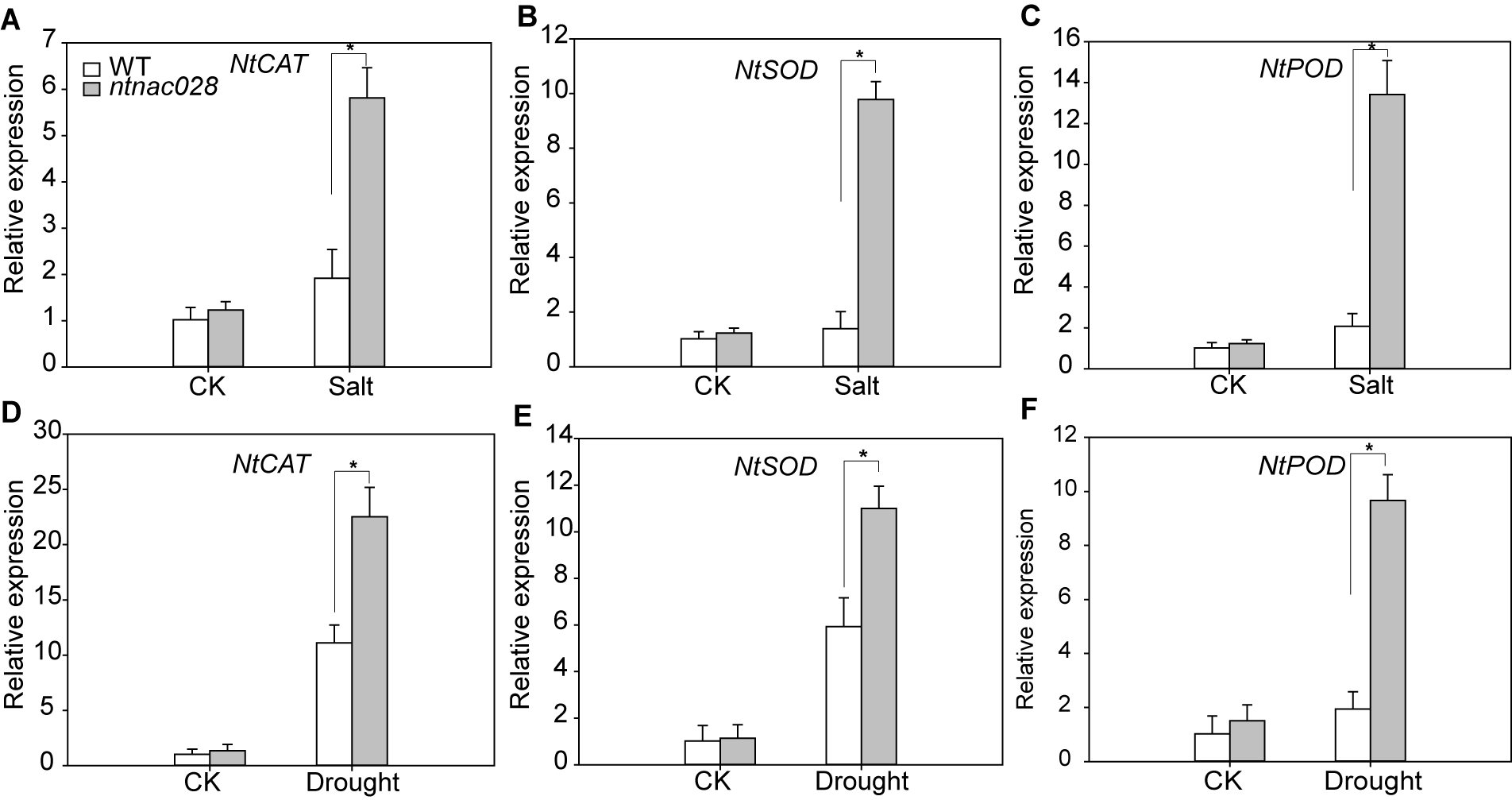

Supplement: Supplementary Figure S6 — Expression patterns of ROS scavenging-related genes in WT and ntnac028 plants in response to salt and drought treatments. [file Image_6.TIF]
