# Supplementary material for: Characterization of NAC transcription factor NtNAC028 as a regulator of leaf senescence and stress responses
Source: Front Plant Sci. 2022 Aug 15;13:941026. doi: 10.3389/fpls.2022.941026 (PMC9421438; doi:10.3389/fpls.2022.941026)
Supplement: Supplementary file 7 [file Data_Sheet_1.docx]

**Supplementary Table S1:** Sequences of primer pairs used for qRT-PCR analysis.

| Name | Forward primer | Reverse primer |
| --- | --- | --- |
| *NtNAC028* | 5’-ATTGCCAAGGGCTTGTTCACTATCT-3’ | 5’-GTGTTTTGGTTGTTAGCGTTGTATG-3’ |
| *NtActin* | 5’-ACCTCTATGGCAACATTGTGCTCAG-3’ | 5’-CTGGGAGCCAAAGCGGTGATT-3’ |
| *NtCP1* | 5’-CAGTGGCTAATCAACCTGTTTCGG-3’ | 5’-ACACCACTTGAATAGAACTGGAAATCG-3’ |
| *NtRBCS* | 5’-CGAAACTCTCTCATACCTTCCCGA-3’ | 5’-CATGGTCCAGTATCTGCCGTCATA-3’ |
| *AtActin* | 5’-TGTGCCAATCTACGAGGGTTT-3’ | 5’-TTTCCCGCTCTGCTGTTGT-3’ |
| *AtSAG12* | 5’-TCCAATTCTATTCGTCTGGTGTGT-3’ | 5’-CCACTTTCTCCCCATTTTGTTC-3’ |
| *NtRD29A* | 5’-GCAGGGAACCAAAATAGGACT-3’ | 5’-CCTCCTTATTGTCGCCTGTAG |
| *NtDREB1B* | 5’-GAATAACCCCAAGAAGCGAGC-3’ | 5’-GAAGGGAAAGTGCCAAGCCAT-3’ |
| *NtRD26* | 5’-GCCACCAGGGTTTCGTTTTT-3’ | 5’-TCGAATTGGGACGACGATGA-3’ |
| *NtNCED3-2* | 5’-TGTCCGAGGATGATTTACCG-3’ | 5’-AAAGCAAATAACTCCCCTGA-3’ |
| *NtSOD* | 5’-CTCCTACCGTCGCCAAAT-3’ | 5’-GCCCAACCAAGAGAACCC-3’ |
| *NtCAT* | 5’-AGGTACCGCTCATTCACACC-3’ | 5’-AAGCAAGCTTTTGACCCAGA-3’ |
| *NtPOD* | 5’-GCTGTTCGACGAGTTGTTAACAG-3’ | 5’-CTCTGGCTGAGTTGTTGTTGG-3’ |
| *AtCAT2* | 5’-TCCCGTCGAGGTATGACCAGGTT-3’ | 5’-CTTGCCAGCTTCTGTCCCAAAGACT-3’ |
| *AtSOD* | 5’-TTGTGTTGTGACGACAAGC-3’ | 5’-ATCAATCTGCTCAAGAACAC-3’ |
| *AtPOD* | 5’-TATGGAAGTGGCGGCTAT-3’ | 5’-ACAATGGACTGAACAATCTCTT-3’ |
| *AtRD29A* | 5’-ATCATCTGGCTGGTTTGGTG-3’ | 5’-AACAACAGTGGAGCCAAGTG-3’ |
| *AtDREB1B* | 5’-TTGGGATGCCGACTTTGTTG-3’ | 5’-TAACTCCAAAGCGACACGTC-3’ |
| *AtRD26* | 5’-ATGTGAATCGGCAGCAGAAC-3’ | 5’-TTGACCCGAAACACCAAACC-3’ |
| *AtNCED3-2* | 5’-ACGCCGTCAAATTCGAACAC-3’ | 5’-AAAACCGGTCGACCCAATTG-3’ |

**Supplementary table 2**: Detailed description of the key cis-element present in NtNAC028 promoter sequences. (Presence of cis-elements on both - & + strand are represented in a single column).

| S.No. | Cis-element | Sequence | ﹢&﹣strand | Organism | Function |
| --- | --- | --- | --- | --- | --- |
|  | ABRE4 | CACGTA | 388+ | Zea mays |  |
|  | AAGAA-motif | GAAAGAA | 1417/1419+ | Avena sativa |  |
|  | ABRE3a | TACGTG | 388- | Zea mays |  |
|  | AE-box | AGAAACAA | 2606+ | Arabidopsis thaliana | part of a module for light response |
|  | ARE | AAACCA | 584/589+  2656- | Zea mays | cis-acting regulatory element essential for the anaerobic induction |
|  | ABRE | ACGTG | 336/396/510/1095/2378+  2377- | Arabidopsis thaliana | cis-acting element involved in the abscisic acid responsiveness |
|  | Box 4 | ATTAAT | 1534/1694- | Petroselinum crispum | part of a conserved DNA module involved in light responsiveness |
|  | CGTCA-motif | CGTCA | 963/1054+  2060/2101- | Hordeum vulgare | cis-acting regulatory element involved in the MeJA-responsiveness |
|  | CARE | CAACTCCC | 916+ | Oryza sativa |  |
|  | GATA-motif | AAGATAAGATT/AAGGATAAGG | 2531+  2775- | Arabidopsis thaliana/Solanum tuberosum | part of a light responsive element |
|  | GARE-motif | TCTGTTG | 244+ | Brassica oleracea | gibberellin-responsive element |
|  | G-box | CACGAC/TACGTG | 661+  329/388/2021/2377- | Zea mays/Arabidopsis thaliana | cis-acting regulatory element involved in light responsiveness |
|  | GC-motif | CCCCCG | 1318+ | Zea mays | enhancer-like element involved in anoxic specific inducibility |
|  | G-Box | CACGTT(G) | 335/509/2377- | Pisum sativum | cis-acting regulatory element involved in light responsiveness |
|  | HD-Zip 1 | CAAT(A/T)ATTG | 1335+ | Arabidopsis thaliana | element involved in differentiation of the palisade mesophyll cells |
|  | I-box | AAGATAAGGCT | 2531+ | Gossypium hirsutum | part of a light responsive element |
|  | LTR | CCGAAA | 879- | Hordeum vulgare | cis-acting element involved in low-temperature responsiveness |
|  | MRE | AACCTAA | 1591- | Petroselinum crispum | MYB binding site involved in light responsiveness |
|  | MYB-like sequence | TAACCA | 1198+ | Arabidopsis thaliana |  |
|  | TC-rich repeats | GTTTTCTTAC | 1798+  1419- | Nicotiana tabacum | cis-acting element involved in defense and stress responsiveness |
|  | TGACG-motif | TGACG | 2060/2101+  963/1054- | Hordeum vulgare | cis-acting regulatory element involved in the MeJA-responsiveness |
|  | WUN-motif | AAATTTCTT | 2296+ | Nicotiana glutinosa |  |
